# Changes in nasal septum morphology after rapid maxillary expansion: a Cone-Beam Computed Tomography study in pre-pubertal patient

**DOI:** 10.1590/2177-6709.25.5.051-056.oar

**Published:** 2020

**Authors:** Giovanni Bruno, Alberto De Stefani, Celeste Benetazzo, Francesco Cavallin, Antonio Gracco

**Affiliations:** 1 Università di Padova, Faculty of Dentistry (Padova, Italy).; 2 Private practice (Solagna, Italy).

**Keywords:** Nasal septum deviation, RPE, Rapid maxillary expansion, Maxillary transverse hypoplasia

## Abstract

**Introduction:**

Nasal septum deviation (NSD) is the most common structural cause of nasal obstruction, affecting around 65-80% of the adult population. Rapid maxillary expansion (RME) is currently used for treatment of maxillary transverse deficiency, but can also influence nasal cavity geometry.

**Objective:**

The present study aimed at evaluating the changes in NSD by using Cone-Beam Computed Tomography (CBCT) scans in pre-pubertal patients treated with RME.

**Methods:**

This retrospective exploratory study evaluated 20 pre-pubertal patients (mean age 10 ± 2 years) who were treated for transverse maxillary constriction with RME and presented mild/moderate NSD as an incidental finding. The outcome measures were NSD tortuosity and area. These measures were obtained from transverse and coronal views of records taken before and after RME treatment. Intra-rater reliability was also assessed with intraclass correlation coefficient.

**Results:**

NSD was mild in thirteen patients (65%) and moderate in seven (35%). NSD tortuosity index did not significantly change over time (mean difference 0.002 mm/year, 95% CI; *p* = 0.58). NSD area did not significantly change over time (mean difference 2.103 mm^2^/year, 95% CI; *p* = 0.38). Intraclass correlation coefficient was 0.73 (95% CI) for NSD tortuosity and 0.84 (95% CI) for NSD area.

**Conclusions:**

NSD tortuosity and area suggested potential changes in NSD with small clinical relevance in pre-pubertal patients who were treated with RME. Additional studies using CBCT scans in larger samples are required to clarify the role of RME in NSD treatment.

## RESUMO

**Introdução:**

O desvio de septo nasal (DSN) é a causa estrutural mais frequente de obstrução nasal, afetando de 65% a 80% da população adulta. A expansão rápida da maxila (ERM), atualmente utilizada para o tratamento da deficiência transversa da maxila, também pode influenciar na geometria da cavidade nasal.

**Objetivos:**

O presente estudo teve como objetivo avaliar, usando tomografia computadorizada de feixe cônico (TCFC), as mudanças no DSN após o tratamento com ERM em pacientes pré-púberes.

**Métodos:**

Esse estudo exploratório retrospectivo avaliou 20 pacientes pré-púberes (idade média de 10 ± 2 anos) com deficiência transversa da maxila tratados com ERM, e que apresentavam DSN de leve a moderado, como um achado incidental. Foram realizadas medições da tortuosidade e área do DSN. Essas medições foram feitas em cortes transversais e coronais das TCFCs pré- e pós-tratamento com ERM. A confiabilidade intraexaminador também foi aferida por meio do coeficiente de correlação intraclasse.

**Resultados:**

O DSN era leve em 13 pacientes (65%) e moderado em 7 (35%). O índice de tortuosidade do DSN não mudou significativamente ao longo do tempo (diferença média = 0,002 mm/ano, IC 95%; *p*= 0,58). A área do DSN não mudou significativamente ao longo do tempo (diferença média = 2,103 mm^2^/ano, IC 95%; *p*= 0,38). O coeficiente de correlação intraclasse foi igual a 0,73 (IC 95%) para a tortuosidade do DSN e 0,84 (IC 95%) para a área do DSN.

**Conclusões:**

Os valores de tortuosidade e da área do DSN sugeriram potenciais mudanças no DSN, mas com pequena relevância clínica, nos pacientes pré-púberes tratados com ERM. Estudos adicionais utilizando TCFC em amostras maiores são necessários para esclarecer o papel da ERM no tratamento do DSN.

## INTRODUCTION

Nasal septum is an osteo-cartilaginous structure forming medial portion of nasal cavity, composed of septal nasal cartilage and perpendicular plate of the ethmoid bone and vomer bone. It is an important functional and esthetic structure for proper nasal respiration because it concurs to regulate airflow through the nose.^1^ A straight nasal septum ensures a laminar airflow allowing the inspired air to be warmed, humidified and cleaned, in order to optimize the alveolar gas exchanges.^1^ ^,^ ^2^ Inversely, a nasal septum deviation (NSD) concurs to nasal obstruction and impaired nasal respiration.^2^ NSD is defined as a deflection from the midline, which can be caused by congenital deformation, traumatic/iatrogenic injury or important nasal infection.^3^ NSD is the most common structural cause of nasal obstruction,^1^ affecting around 65-80% of the adult population.^4^ ^,^ ^5^ Although it is often physiological, NSD may require septoplasty surgical operation when it causes a severe grade of obstruction (≥ 16°).^6^ ^-^ ^8^ This situation can also negatively affect the midfacial development in growing patients.^9^ NSD is associated with many skeletal and dental problems, such as Class II malocclusion, increased overjet, retrognathic maxilla and mandible, increased anterior facial height, maxillary transverse deficiency associated with crossbite, high arched palate, low tongue posture and incompetent lips.^1^ ^,^ ^2^ ^,^ ^10^

The maxillary transverse deficiency is one of the most frequent problems in the craniofacial complex, causing usually monolateral or bilateral crossbite, crowding, high arched palate and mouth breathing.^11^ Therefore, it is very important to identify and resolve this problem in children and adolescents. The most effective treatment is increasing maxillary width by using rapid maxillary expansion (RME), which is as a safe, reliable, tolerable, simple and predictable orthopedic procedure.^12^ ^,^ ^13^ RME treatment aims to coordinate skeletal bases by opening the midpalatal suture, avoiding dental orthodontic effects as much as possible.^14^

Maxillary bones form the anatomical base of the nasal cavity, thus RME can influence nasal cavity geometry.^12^ ^,^ ^15^ A recent systematic review^1^ included only two studies with heterogeneous participants and results: Farronato et al.^15^ reported NSD reduction in 94% of cases treated with RME, while Altug-Atac et al.^16^ did not found any changes in NSD. The main weakness of these studies is the measurement using posteroanterior radiographs. Aziz et al.^17^ evaluated NSD using Cone-Beam Computed Tomography (CBCT) scans and did not find any significant differences in NSD after treatment with RME in adolescents. However, using RME should be preferred before the pubertal peak of growth (CS1-CS3^18^ ^-^ ^20^) in order to achieve orthopedic rather than dental effects.^21^

Thus, the present study aimed at evaluating the changes in NSD by using CBCT scans in pre-pubertal patients treated with RME. 

## MATERIAL AND METHODS

### Study design

This is a retrospective exploratory study. The study was conducted according to the Helsinki Declaration principles and patients gave their consent to have their data collected for scientific purposes. The study was approved by the local Ethics Committee of *Azienda Ospedaliera di Padova* (protocol # 41648).

### Patients

Twenty patients treated with RME for maxillary transverse deficiency were included in the study (mean age 10 ± 2 years). The inclusion criteria were: pre-pubertal patients (CS1-CS3^18^ ^-^ ^20^); skeletal maxillary transverse constriction with or without posterior crossbite; no previous orthodontic treatment; availability of pre- and post-treatment CBCT; NSD from mild to severe. Patients with congenital or dental anomalies and previous orthodontic treatment were excluded. NSD was discovered as an incidental finding in pre-treatment CBCT scans. Authors considered a control group, but it was not possible to collect pre- and post-treatment CBCT in patients without need of RME, for ethical limits. The CBCT scans were taken with the patient’s head oriented in the same Cartesian plan.

### Intervention

Each patient was treated with a Haas expander. The protocol of activation consisted in activation of the screw one-quarter turn twice a day for a variable period, depending on transverse constriction severity. Then RME expander was left in place for six months, for passive retention. 

### Image analysis

**Figure 1:**
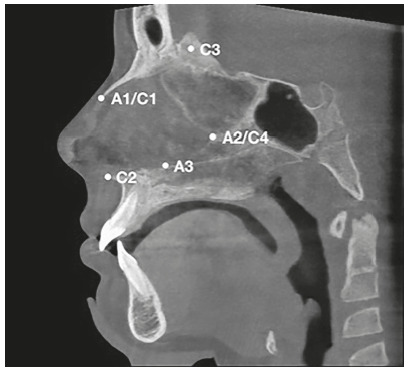
Landmarks on sagittal view.

**Figure 2:**
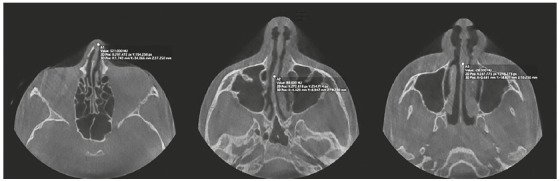
Landmarks on axial view.

**Figure 3:**
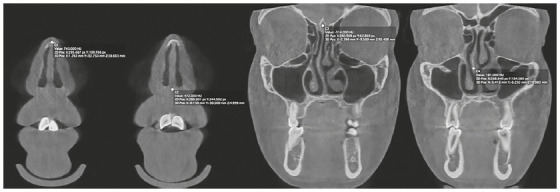
Landmarks on coronal view.

NSD was identified analyzing transverse and coronal views of CBCT records taken before RME treatment.^2^ NSD was considered mild (≤ 8°), moderate (from 9° to 15°) or severe (≥ 16°).^6^ ^,^ ^7^ All CBCT scans were taken with Soredex Scanora 3D (PaloDEx, Tuusula, Finland) before the beginning of the treatment (T_1_) and after at least 12 months following the treatment (T_2_). Images were converted into DICOM format, with a voxel size of 0.25 mm, and uploaded to Horos Project (v. 2.4.1, 64 bit) which is a free, open source medical image viewer (*https://horosproject.org/about/*). Landmarks were identified in the 3D viewer and 2D orthogonal mode in Horos Project for each patient in sagittal view, according to previous studies^16^ ^,^ ^22^ (Figs 1 to 3). These landmarks were used to identify three axial (A1, A2, A3) and four coronal DICOM landmarks (C1, C2, C3, C4) for each patient at each time point. The axial landmarks included: the anterior point of nasal bone (A1), the junction of perpendicular plate of ethmoid bone and vomer (A2), and the midway point between A2 and C2 (A3). The coronal view included: the anterior point of nasal bone (C1), the anterior nasal spine (C2), the midpoint of crista galli (C3), the junction of perpendicular plate of ethmoid bone and vomer (C4). All measurements were repeated three times with seven days interval. Fourteen images were evaluated in each patient and were transferred to Matlab (MathWorks R2017b, Natick, Massachusetts) for NSD analysis. Data on NSD from Matlab were transferred to statistical software for data analysis.

### Outcome measures

The outcome measures were NSD tortuosity and NSD area. NSD tortuosity was calculated as the ratio of length of the curve to the length of an imaginary line in the midsagittal plane, according to previous studies^16^ ^,^ ^22^. NSD area was calculated as the integral from the curve to an imaginary line in the midsagittal plane, according to previous studies.^16^ ^,^ ^22^

### Statistical analysis

Continuous data were expressed as mean and standard deviation (SD). Intra-rater reliability was assessed with intraclass correlation coefficient (ICC) and 95 per cent confidence interval (CI).^23^ The average of the three measurements at each time point (T_1_ and T_2_) was calculated for each subject and used for further analysis. Given the different length of follow-up among patients, the variations in NSD tortuosity and in NSD area were calculated as the difference over time (i.e., T_2_-T_1_) divided by the length of follow-up in each subject. Variations over time were evaluated using paired Student *t*-test and expressed as mean difference (MD) with 95 per cent confidence interval (95% CI). Association of NSD variations over time with age and width of expansion was evaluated using Pearson correlation coefficient. All tests were 2-sided and a *p*-value of less than 0.05 was considered statistically significant. Statistical analysis was performed using R 3.3 (R Foundation for Statistical Computing, Vienna, Austria).^24^

## RESULTS

The study included 20 pre-pubertal individuals: NSD was mild in 13 patients (65%) and moderate in 7 patients (35%). Crossbite was observed in six patients (30%). Median width of expansion was 6.4 mm (SD = 0.8). Mean follow-up was 2.5 years (SD = 0.6).

**Figure 4:**
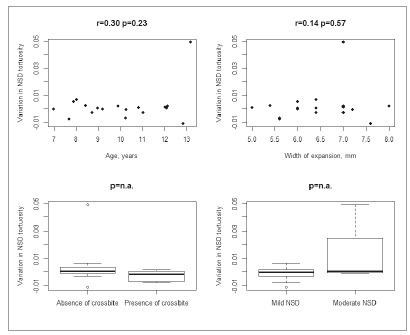
Variation in NSD tortuosity (mm/year) according to age, width of expansion, presence of crossbite and NSD severity (n.a.= not available).

**Figure 5:**
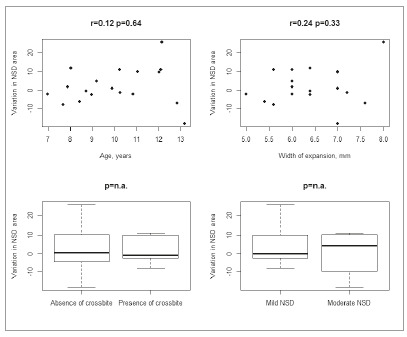
Variation in NSD area (mm^2^/year) according to age, width of expansion, presence of crossbite and NSD severity (n.a.: not available).

ICC was 0.73 (95% CI = 0.60 to 0.86) for NSD tortuosity and 0.84 (95% CI = 0.75 to 0.92) for NSD area. NSD tortuosity did not significantly change over time (MD = 0.002 mm/year, 95% CI -0.005 to 0.008; *p*= 0.58). NSD area did not significantly change over time (MD = 2.103 mm^2^/year, 95% CI -2.283 to 7.039; *p*= 0.38). Pre-treatment age and width of expansion were not associated with NSD tortuosity or NSD area (Figs 4 and 5).Graphical summary of NSD tortuosity and NSD area according to presence/absence of crossbite and NSD severity (mild/moderate) is shown in Figures 4 and 5. The limited sample size did not allow any meaningful statistical comparisons regarding crossbite and NSD severity.

## DISCUSSION

NSD is the most common structural cause of nasal obstruction and it is a prevalent problem among the general population.^1^ ^,^ ^4^ ^,^ ^5^ Surgical treatment is usually performed in patients reporting symptomatic nasal obstruction associated with NSD, while a deviated septum without other symptoms is not an indication for septoplasty.^8^ ^,^ ^25^ RME is currently used for treatment of maxillary transverse deficiency, but can also influence nasal cavity geometry because maxillary bones form the anatomical base of the nasal cavity.^12^ ^,^ ^15^ To our knowledge, few data on the effect of RME on NSD are available in literature.

The present data did not show any significant variations in NSD at long-term follow-up in pre-pubertal patients treated with RME. Although the limited sample size could affect statistical significance, the estimates of tortuosity and area nevertheless suggested potential changes in NSD with small clinical relevance. The present findings were in agreement with a previous study evaluating NSD in adolescents by using CBCT scans.^17^ Aziz et al.^17^ did not report any significant effect of RME in adolescents who were treated for mild to severe NSD. Other two studies investigated RME in NSD by using posteroanterior radiographs.^15^ ^,^ ^16^ Farronato et al.^15^ reported NSD reduction in children aged 5-9 years treated with RME, while Altug-Atac et al.^16^ confirmed no effect of RME in NSD in adolescents. Available studies in literature present high heterogeneity regarding included participant age (children and adolescents), deviation degree (from mild to severe) and assessment tool (CBCT scans or posteroanterior radiographs). In addition, posteroanterior radiographs do not allow a good evaluation of anatomical measurements because of the overlap of the different anatomical structures. 

CBCT scans are among the suggested diagnostic tools for NSD because it provides an accurate evaluation of anatomical measurements and allows a comprehensive assessment of deviation-related respiratory problems.^2^ The present data showed good reliability of CBCT scans in identification of anatomical landmarks, in agreement with Aziz et al.^17^

RME is a beneficial procedure in the resolution of maxillary constriction but also in the treatment of nasal respiratory problems.^12^ ^,^ ^26^ The opening of the midpalatal suture allows significant widening of maxillary bone and increasing of intranasal cavity. Moreover, the increase in nasal cavity width is associated with lowering of the palatal vault that reduces nasal resistance, ensuring a better nasal airflow.^26^ ^-^ ^28^ This effect leads to a marked improvement in nasal breathing with also a remarkable stability of the increments of nasal dimensions in the long-time period.^27^ ^,^ ^28^ Such improvements are likely to be associated with the increase in area and volume of the nasal cavities rather than with changes in the nasal septum morphology. 

The strengths of the present study included NSD evaluation by using CBCT scans and the inclusion of pre-pubertal patients. CBCT scans can provide more reliable identification of landmarks with respect to posteroanterior radiographs.^1^ Moreover, using RME should be preferred before the pubertal peak of growth (CS1-CS3^18^ ^-^ ^20^) in order to achieve more effective long-term orthopedic effects.^21^ Although the mechanism regulating the development process has not been fully clarified, the septal cartilage has been suggested to play a main role in the down-forward repositioning of the nasomaxillary complex^29^ together with the soft tissue stimulus.^30^

This study has some limitations. First, it is a retrospective study and post-treatment evaluation was available at different time points. However, we calculated changes in NSD divided by the length of follow-up in each patient. Second, there was no control group, because RME is currently used for maxillary transverse deficiency and all patients with maxillary transverse deficiency were treated with RME. Third, the limited sample size did not allow any meaningful statistical comparisons according to presence of crossbite and NSD severity. These limitations are suggestions that could be considered for further researches.

## CONCLUSIONS

NSD tortuosity and area suggested potential changes in NSD with small clinical relevance in pre-pubertal patients who were treated with RME. Additional studies using CBCT scans in larger samples are required to clarify the role of RME in NSD treatment.
